# Can Hyaluronic Acid Combined with Chondroitin Sulfate in Viscosupplementation of Knee Osteoarthritis Improve Pain Symptoms and Mobility?

**DOI:** 10.3390/biom14070832

**Published:** 2024-07-11

**Authors:** Augustin Dima, Magda Dragosloveanu, Andreea Ramona Romila, Alexandru Cristea, Georgiana Marinică, Alexandru-Tiberiu Dănilă, Alexandru Mandici, Daniel Cojocariu, Robert-Alexandru Vlad, Adriana Ciurba, Magdalena Bîrsan

**Affiliations:** 1 Department of Medical Rehabilitation-Orthopedics and Traumatology, National Institute of Rehabilitation, Physical Medicine and Balneoclimatology, 2 Sf. Dumitru Street, Sector 3, 030167 Bucharest, Romania; draugustindima@yahoo.com (A.D.); dr.alexandrucristea@gmail.com (A.C.); 2 Department of Rehabilitation, University of Medicine and Pharmacy “Carol Davila”, 8 Eroii Sanitari Street, Sector 5, 050474 Bucharest, Romania; magda.dragosloveanu@umfcd.ro; 3 Department of Rehabilitation, Physical Medicine and Balneology II, National Institute of Rehabilitation, Physical Medicine and Balneoclimatology, 2 Sf. Dumitru Street, Sector 3, 030167 Bucharest, Romania; andreea.romila@gmail.com; 4 Medical and Pharmacovigilance Department, Rompharm Company SRL, 1A Eroilor Street, 075100 Otopeni, Romaniaalexandru-mandici@email.umfiasi.ro (A.M.); daniel-cojocariu@email.umfiasi.ro (D.C.); 5 Department Pharmaceutical Sciences II, Faculty of Pharmacy, Grigore T Popa University of Medicine and Pharmacy Iasi, 16 Universitatii Street, 700115 Iasi, Romania; 6 Department of Pharmaceutical Technology and Cosmetology, Faculty of Pharmacy, George Emil Palade University of Medicine, Pharmacy, Science, and Technology, 38 Gheorghe Marinescu Street, 540142 Targu Mures, Romaniamagdalena.birsan@umfiasi.ro (M.B.); 7 Department of Pharmaceutical Industry and Pharmaceutical Biotechnologies, Grigore T Popa University of Medicine and Pharmacy Iasi, 16 Universitatii Street, 700115 Iasi, Romania

**Keywords:** hyaluronic acid intra-articular, chondroitin sulfate, viscosupplementation, knee osteoarthritis, pre-filled syringe, infiltrations

## Abstract

The objective of the present study was to assess the effect of intra-articular Hyaluronic acid (HA) and Chondroitin sulfate (CS) supplementation (Hialurom^®^ Hondro (HH)) on pain symptoms and joint mobility. In total, 60 mg/mL sodium hyaluronate and 90 mg/mL CS were administered to 21 patients (17 females and 4 males) respecting the in-force requirements, excluding patients with some specific comorbidities. In addition to the clinical study (where the pain intensity (severity) and joint mobility were assessed), rheological characterization was conducted evaluating the following parameters: elastic modulus (G′), loss modulus (G″) oscillatory frequency (fc) at 0.5 Hz and 2.5 Hz, crossover frequency (fc), relaxation time (λ) where it was noticed that the addition of chondroitin sulfate (CS) to sodium hyaluronate (SH) significantly enhances and improves the viscoelastic properties, particularly at higher shear frequencies. A significant decrease in pain intensity felt by the subjects was found, from 7.48 (according to Wong–Baker scale)—pain close to 8 (the patient is unable to perform most activities), to more reduced values of 5.86—at 6 weeks after injection, 4.81—at 3 months after injection, and 5.24—at 6 months after injection, improvements in symptoms was fast and durable. Data related to the evolution of joint mobility show that at 6 weeks after injection, the mobility of joints increased by 17.8% and at 6 months by 35.61%. No serious adverse events were reported with undesired effects so that they would impose additional measures. Better resistance to enzymatic degradation and free radicals could be expected from the synergic combination of sodium hyaluronate and chondroitin sodium sulfate, this having a special importance for the patients, granting them the ability to perform more ample movements and reducing dependency on attendants, thus increasing quality of life.

## 1. Introduction

Osteoarthritis (OA) represents the major cause of disability in older adults, leading to loss of function, pain, and a decreased quality of life. Estimates suggest that approximately 528 million people worldwide are affected by OA which affects sleep quality, mood, and participation in everyday life, limiting a person’s ability to self-manage other conditions, such as diabetes and hypertension [1]. The disease most commonly affects the joints in the knees, hands, feet, and spine and is relatively common in shoulder and hip joints. While knee OA is related to aging, it is also associated with a variety of risk factors, such as obesity, lack of exercise, occupational injury, and trauma [2,3]. Over the course of OA, articular cartilage gets severely degraded leading to constant or intermittent pain of different intensities, frequently associated with stiffness, swelling, and joint mobility loss. Although most changes occur in the cartilage, the entire joint is affected, including ligaments, synovium, and subchondral bone. Initially, chronic overloading and impaired biomechanics were thought to be the main causes of OA, which subsequently led to joint articular destruction and inflammation [4,5]. However, OA seems to be triggered by a complex process involving metabolic and inflammatory factors, such as active synovitis and systemic inflammation [6]. Osteoarthritis (OA) is a prevalent condition, affecting a significant portion of the population. While the incidence increases with age, with the highest rates between 55 and 64 years old for knee OA, it is important to note that more than half of individuals with symptomatic knee OA are younger than 65 years old. Furthermore, women are disproportionately affected by OA, with 78% of adults with OA occurring in females. An even more disproportionate share of 18% of the population age 65 and older have OA (43%). This compares to 46% with osteoarthritis in 34% of the population aged 45 to 64 years [7]. This prevalence is likely to rise in the coming years, as the global population ages rapidly. According to World Health Organization (WHO) in 2020, there were already 1 billion people aged 60 or over worldwide, and this figure is projected to reach 1.4 billion by 2030, representing one in six people globally [8]. Therefore, OA represents a growing public health concern, demanding continued research into prevention, management, and treatment strategies.

OA management should be focused initially on non-pharmacological interventions such as weight loss and exercise, followed by pharmacologic interventions such as non-steroidal anti-inflammatory drug (NSAIDs) (systemic and/or topical) intra-articular (IA) corticosteroids and/or hyaluronic acid (HA), whereas surgery is deemed as last resort solution [9,10,11,12,13]. Pharmacological interventions mainly focus on long-term pain alleviation by using either selective or non-selective NSAIDs. Although efficient in alleviating OA-related pain, their long-term use results in various adverse events, including gastric ulcers, bleeding, and renal failure [14,15]. Avoiding systemic adverse events, NSAIDs imply that alternative therapies such as viscosupplementation should be considered. Although invasive, intraarticular (IA) HA administration is regarded as generally safe and has targeted results, intending to reinforce synovial fluid viscoelasticity [16,17]. Pain and disability management on a chronic or relapsing course may ask for polymodal therapies, such as physical agents, platelet-rich plasma injections, and botulinum toxin administration [18].

Particularly regarding knee OA management by IA-HA viscosupplementation, there is no consensus between national and international guidelines in recommending IA-HA administration in OA-affected joints. The American College of Rheumatology recommends it for patients with no response to conventional treatments and patients with contraindications to surgery [19].

HA represents a major component of synovial fluid and cartilage. Due to its viscoelastic and rheological properties, it decreases articular friction and protects soft tissue against trauma, being responsible for cushioning and lubricating synovial joints. However, its viscosity decreases as shear forces increase, an essential behavior for lubricating joints during rapid joint movement. Conversely, high viscosity at low shear forces is required for joint stabilization [13,20]. Chondroitin sulfate (CS) has a gel-like structure and plays a major role in maintaining the structural integrity of tissues by linking to monomers with high molecular weights, being mainly located around the cartilage of the joints. Furthermore, CS inhibits extracellular proteases involved in connective tissue metabolism, and cartilage cytokine production and induces articular chondrocytes apoptosis [21]. More than 40 years have passed since the first FDA-approved IA injection of HA as sodium hyaluronate (SH) for the treatment of pain in patients with knee OA in 1997. Its approval was based on positive outcomes during clinical trial investigation in patients with knee OA and safety regarding administration [22]. Later it has been noted that SH and CS association in aqueous solution seems to increase solution’s viscosity. One potential explanation resides in the increased viscosity when associating CS and SH via hydrogen bonds between N-acetylamino groups, increasing their molecular size, while also having a crosslinking tendency of long fractions, further leading to an increase in viscosity [20,21,23]. Based on these observations, CS could be used to improve HA rheological properties to significantly improve synovial fluid properties and enhance lubrication. Binding to core proteins through N and O linkages leads to aggregates of monomers with high molecular weights. The proteoglycan aggregate has viscoelastic and hydration properties and an ability to interact with the adjacent tissue through electric charges leading to cartilage tissue protection. Non-animal SH and its natural crosslinking with CS leads to increased bioavailability, with mechanical and physicochemical properties similar to human synovial fluid. These biopolymers act as a scaffold, binding other matrix molecules including aggrecan, being involved in several important biological functions such as cell adhesion and cell motility regulation, cell differentiation and proliferation, and providing biomechanical properties [21]. Researchers identify HA as a major supplementation for a wide range of degenerative joint diseases, particularly hemophilic arthropathy which shares many features with osteoarthritis [23,24].

Considering all the aspects, the objective of the present study was to assess after a single injection the effect of intra-articular HA-CS supplementation on pain symptoms and joint mobility, as well as its safety. In the literature, there are very few or incomplete data, which supports and highlights the importance of this study.

## 2. Materials and Methods

### 2.1. Materials

**Figure 1:**
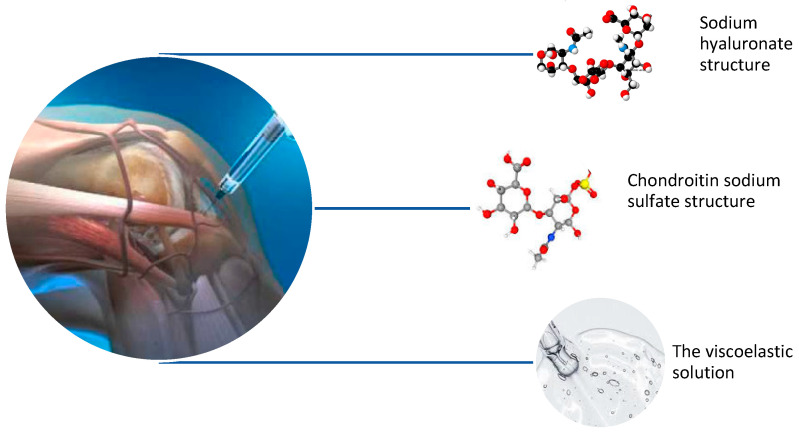
The knee injection of HH by a specialist physician to patients with stage II or stage III KOA: the best approach is the path of least obstruction and maximal access to the synovial cavity, which could be superolateral, superomedial, or anteromedial/anterolateral.

In the present study, a commercially available viscoelastic solution (Hialurom Hondro^®^, Rompharm Company S.R.L., Otopeni, Romania) was investigated. HH is a sterile, isotonic, viscoelastic solution containing two highly purified and natural crosslinked biopolymers in phosphate buffer, sodium hyaluronate with an average molecular weight of 3000 kDa and chondroitin sodium sulfate with an average molecular weight of 25 kDa [25]. Hialurom Hondro^®^ as biomatrix consists of sodium hyaluronate (SH) 60 mg/3 mL derived from bacterial fermentation and chondroitin sodium sulfate (CS) 90 mg/3 mL produced from bovine tracheal cartilage. The viscoelastic solution was administered intra-articular by an orthopedic specialist to patients with stage II or stage III knee osteoarthritis (KOA) (Figure 1).

### 2.2. Patient Enrollment and Study Protocol

This was a monocentric, open-label, confirmatory, randomized, and non-controlled study, involving patients with diagnosed osteoarthritis (OA). Patients’ enrollment was at the National Institute of Rehabilitation, Physical Medicine, and Balneoclimatology (Bucharest, Romania). The study period was between March 10 and November 7 and for inclusion criteria, eligible patients were those between 18 and 75 years old and diagnosed with stage II and III OA, according to the Kellgren–Lawrence scale [25]. Exclusion criteria were patients diagnosed with septic arthritis, those who suffered from traumatic events of the joint intended to treat, those with Paget’s disease, gout, major dysplasia, Wilson’s disease, acromegaly, ochronosis, hemochromatosis, Ehlers–Danlos syndrome, Charcot arthropathy, hypo-/hyper-parathyroidism, active synovitis, rheumatoid arthritis, dermatological conditions at the injection site, and patients who underwent arthroscopy at least 1 year before or received IA steroid injection or HA at least 6 months before investigation. There were no patients from vulnerable groups (paediatrics, pregnant or lactating women, patients with hepatic and/or renal impairment, or populations with specific racial and/or ethnic origins).

The primary endpoints of the study were pain scores and joint mobility measured using the following parameters: pain intensity felt by participants was quantified using the Wong–Baker scale for rating pain intensity [26]. Lequesne index for lower limb arthrosis was used for pain severity evaluation [27]. Joint mobility was assessed by measuring movement amplitude in all directions and is rather an expression of the mobilization mode of a segment than a degree of movement measurement. For a better assessment of its evolution, it was considered that the sum of joint mobility values before injection was 100% and the percentage value was calculated for the sums of corresponding values at weeks 6, 12, and 24, respectively. Joint mobility was assessed with a transparent goniometer (20 cm). As a secondary endpoint, quantifying adverse incident occurrence. Initial clinical evaluation took place before performing the injection. Follow-up timepoints for evaluations were at weeks 6, 12, and 24, assessing the aforementioned parameters at each visit.

Prior to HA-CS injection, the injection site was properly disinfected according to clinical settings procedures. After disinfection, any fluid accumulation in the joints was removed by arthrocentesis. The volume of HA and CS solution for injection was adjusted according to the joint size and IA space of each participant to avoid overfilling. A single IA injection (20 mg/mL SH and 30 mg/mL CSNa) was administered to each patient after initial evaluation.

This study was designed in accordance with the guidelines of the Declaration of Helsinki and standard EN ISO 14155, Clinical investigation in human subjects—Good Clinical Practice [28]. The protocol was approved by the Ethics Committee of the National Institute of Rehabilitation, Physical Medicine and Balneology, Bucharest, Romania (no. 2288). Before enrolling, participants involved in the study were properly informed by the medical staff involved in the investigation and gave their informed consent. The medical device was administered intra-articulary by an orthopedic specialist.

### 2.3. Rheological Parameters of Viscosupplement

Rheological parameters for the viscosupplement were obtained with a Malvern Kinexus Pro+ Rheometer with cone-plate geometry (40 mm plate diameter and 1° cone angle) at 25 ± 0.01 °C or physiological temperature 37 ± 0.01 °C. The tested samples were equilibrated for ten minutes at working temperature before use, each test run was duplicated with a fresh sample. Frequency sweep measurements (G′ and G″ as a function of frequency) were performed in the mode oscillation-controlled deformation (i.e., 0.2% deformation kept constant). The frequency sweep range was 0.01–10.0 Hz.

The parameters collected, e.g., elastic modulus (G′) and loss modulus (G″) as a function of oscillatory frequency (fc), were plotted for identifying viscoelastic profiles and reported as elastic modulus (G′) and loss modulus (G″) corresponding to the transition points 0.5 Hz (representative for walking) and 2.5Hz (representative for running), crossover frequency (fc) defined as the frequency at which G′ = G″, relaxation time (λ (s) meaning the time it takes to recover to its original state following deformation estimated as the inverse of the oscillating frequency at which the elastic modulus (G′) equals loss modulus (G″). The frequency sweeps were performed at strain amplitudes that were determined to be in the linear viscoelastic range [29].

Additional rheological property related to HA-based formulation was considered the percentage of elasticity, i.e., the proportion of elasticity in HA-based formulation, as a function of frequency, calculated by applying 100 × G′/(G′ + G″). Reported values will be related to the reference frequencies 0.5 and 2.5 Hz, by comparison, HA-based formulations with or without chondroitin sulfate. Viscosity measurements depending on shear rate were performed in the dynamic mode viscosity (rotational)—controlled shear by varying shear speed within 0.01–10.00 s^−1^. A 20 mg/mL SH-only viscosupplement served as the control for the comparison of rheological parameters at 25 ± 0.01 °C.

### 2.4. Statistical Analysis

As there was no control group, statistical analysis was mainly descriptive. Measured parameters were expressed as mean ± SD. The admitted error limit for null hypotheses tests was r = 1%. Significance level thresholds for the alternative hypothesis regarding pain intensity, Lequesne index, and joint mobility were α = intense, 0.25, and 0.5, respectively. The average rate of joint mobility variation and joint mobility dispersion were calculated. For the accuracy measurement, Pearson’s coefficient of skewness was calculated. For statistical power, a unilateral *t*-test for independent samples was calculated. A *p* < 0.05 was considered significant.

## 3. Results

A total of 21 patients met the study protocol criteria. As depicted in Table 1, 4 males (19.1%) and 17 females (80.9%) were enrolled in the study (age 61.8 ± 8.2 years). The mean age of OA was 7.9 ± 3.6 years. All enrolled patients completed the study.

### 3.1. Pain Intensity and Severity

Prior to injection, pain was the predominant symptom reported (100% of patients), followed by crackles (66.66% of patients), decreased mobility (52.38%), and impairment of walking (47.64%).

**Figure 2:**
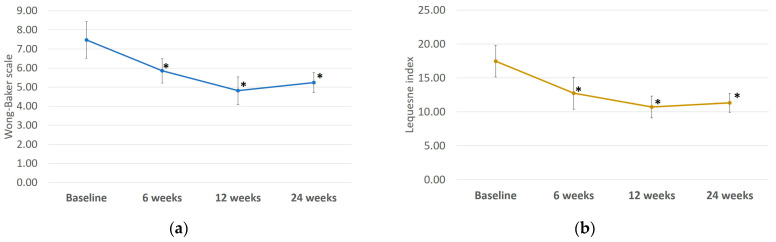
Significant reduction in pain intensity (**a**) (* *p* < 0.001) and pain severity (**b**) (* *p* < 0.001) at weeks 6, 12 and 24 vs. baseline.

Baseline values for pain intensity and severity were pre-injection measurements (7.48 ± 0.96 and 17.48 ± 2.34 for the Wong–Baker scale and Lequesne index, respectively). Concerning the primary endpoint of the study, a significant improvement in the pain intensity score has been noted at follow-up visits (*p* < 0.001) compared to baseline. The average pain intensity decreases at weeks 12 and 24 were 4.81 ± 0.73 and 5.24 ± 0.53, respectively (Figure 2a). Although the latter shows pain with strong discomfort, when it is compared to baseline (7.48 ± 0.96) shows an improvement in patients’ quality of life. Analysis showed a significant reduction in pain severity at follow-up visits (*p* < 0.001) compared to baseline (Figure 2b).

Although significant improvement in severity scores have been noted, the Lequesne index at baseline (17.48 ± 2.34) suggests an extremely severe OA, condition preserved at week 6 (12.76 ± 2.35) and weeks 12 and 24 (10.71 ± 1.61 and 11.33 ± 1.39, respectively) (Figure 2).

### 3.2. Joint Mobility

**Figure 3:**
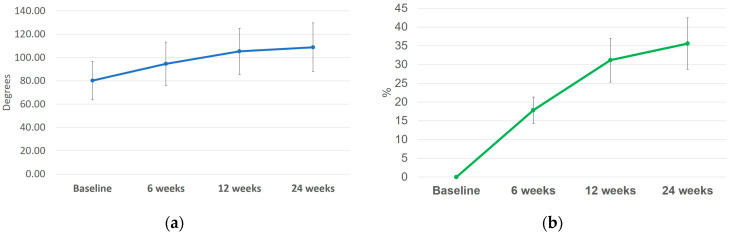
Joint mobility as measured in degrees (**a**) (mean ± SD) and percent (%) increase in joint mobility (**b**) (mean ± SD) at weeks 6, 12, and 24 compared to baseline.

An increase in joint mobility has been noted at weeks 6, 12, and 24 by 17.8 ± 3.51%, 31.5 ± 5.86%, and 35.61 ± 6.85%, respectively, compared to baseline (Figure 3b), with special importance regarding patient movement, as they were allowed to perform higher amplitude movements (Figure 3).

**Figure 4:**
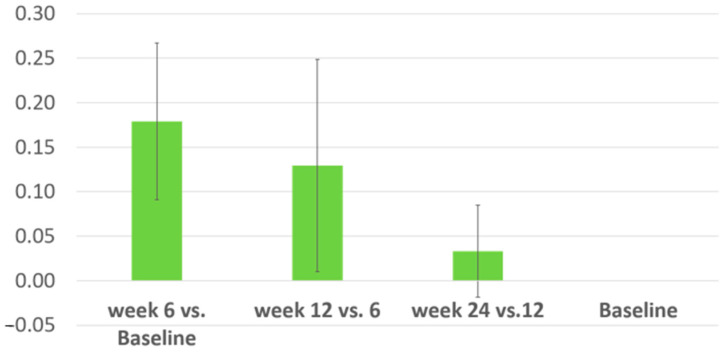
The average rate of joint mobility variation (average ± SD).

The average rate of variation of joint mobility was calculated for each participant at weeks 6, 12, and 24 and compared to baseline. Weeks 6 and 12, respectively, showed positive and towards zero values, meaning an increasing trend in their mobility after HA and CS injection (Figure 4).

To assess data collection precision, joint mobility dispersion and Pearson’s coefficient of skewness were calculated. The calculated Pearson’s coefficient of skewness showed a left, positive skew in all situations, suggesting appropriate data recording (Table 2).

A unilateral *t*-test for independent samples was used to calculate the statistical significance of knee joint mobility measurements. Results showed a significant difference between knee joint mobility at baseline and at follow-up (*p* < 0.001).

### 3.3. Rheological Measurements of Viscosupplement

The rheological parameters of HH at 25 ± 0.01 °C and 37 ± 0.01 °C at 0.5 Hz frequency which are representative for walking and 2.5 Hz which is representative for running are shown in Table 3. The rheological performance of SH (20 mg/mL) and CS (30 mg/mL) formulation vs. another SH formulation without CS (defined as Control, 20 mg/mL SH only) as viscosupplements presented distinctive viscoelastic properties with non-Newtonian behavior, which can enhance and improve viscoelastic properties when shear frequencies are increased (e.g., from walking to running).

**Figure 5:**
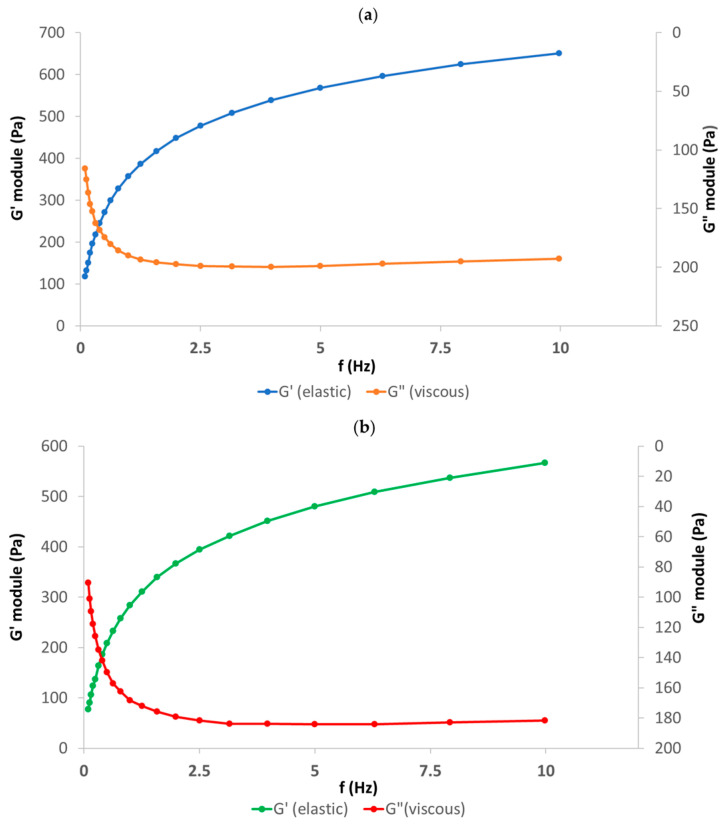
Visco-elasticity profile of HH in a commercial batch at 25 ± 0.01 °C (**a**) and 37 ± 0.01 °C (**b**).

**Figure 6:**
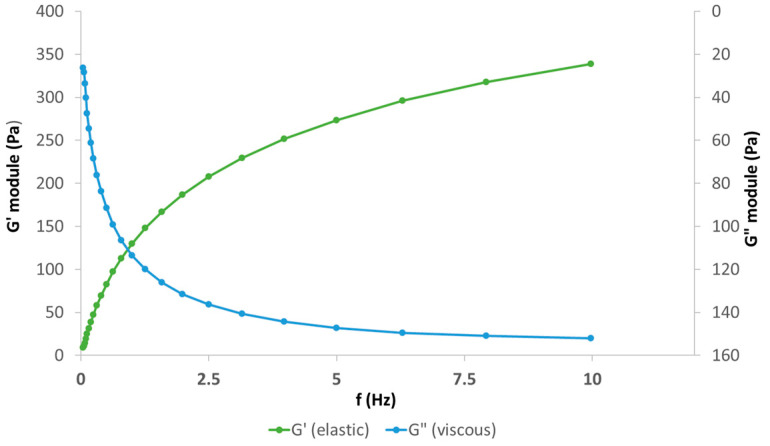
Visco-elasticity profile of positive control commercial batch of HA-hydrogel polymer solution at 25 °C.

Oscillatory frequency provides additional information about the structure–property relationship of biopolymer solution (i.e., sodium hyaluronate and chondroitin sulfate) as it is used to determine the viscous and elastic properties of the sample. By adding chondroitin sulfate to sodium hyaluronate the high-frequency response is defined by the elastic modulus, and the crossover frequency shifts toward lower frequencies or larger relaxation times. Furthermore, at the entanglement region, the elastic modulus reaches a plateau at which point it is independent of frequency and behaves more like an elastic solid (Figure 5 and Figure 6).

**Figure 7:**
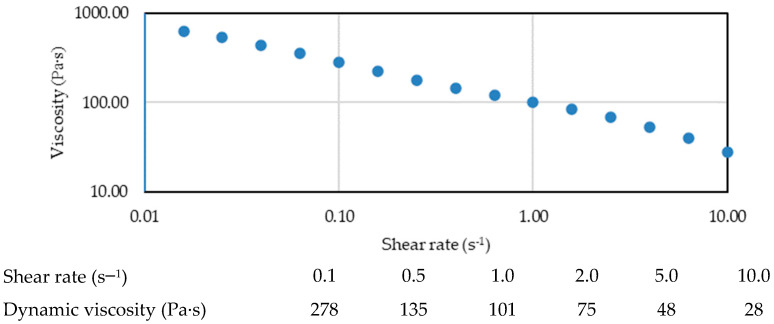
Dynamic viscosity profile for HH commercial batch.

HH showed a robust shear thinning response at high shear rates (Figure 7), relaxation times in the order of seconds 5 ÷ 10 s (Table 4), a flattening of the G′ beyond the crossover frequency, and G″ within and a wider range of linear viscoelastic response dominated by the elastic component.

## 4. Discussion

The purpose of this study was to assess the performance and safety of viscoelastic solution combining Sodium Hyaluronate (20 mg/mL) and Chondroitin Sodium Sulfate (30 mg/mL), administered by IA injection in knee OA patients. All enrolled patients received a single injection at baseline, after initial clinical evaluation, and were assessed at three follow-up timepoints (6, 12, and 24 weeks). Although pain with strong discomfort was considered at baseline, pain intensity and severity decreased significantly at weeks 6, 12, and 24 vs. baseline (*p* < 0.001).

The mean joint mobility at weeks 6, 12, and 24 showed an increase (by 17.8%, 31.5%, and 35.61%, respectively) when compared to mean joint mobility at baseline, while the mean Pearson’s coefficient of skewness showed a left, positive skew for all situations. Unilateral *t*-test showed a significant difference between knee joint mobility at baseline and at follow-up (*p* < 0.001). The results obtained were consistent with previous research conducted by other authors [26,27,29,30,31,32,33], although differences were noted in IA-HA posology between our study protocol and others.

In this study protocol, an HA-CSNa injection was administered once at the beginning of the study, whereas other studies administered one or more injections weekly for the duration of the study. Similar to us, Henrotin et al. (2012) noted an improvement in pain intensity and functional impairment, quantified by the Lequesne index. Significant decreases in pain intensity were similarly noted, at weeks 6 and 12 (*p* = 0.0008 and *p* = 0.0042, respectively) after injection, although no data were recorded at week 24, as in our study. Injections were administered once a week (days 0, 7, and 14 of the study) and followed up at weeks 7 and 14 [30].

Consistent results with which ones obtained through this study were noted in a randomized, blind observer, parallel trial, where a significant improvement in favor of the HA-treated group vs. placebo was noted in Lequesne index at week 5 (*p =* 0.03), with persistent results at week 8 (*p =* 0.0431) and at week 16 (*p =* 0.0528), but with no difference at week 24. The VAS score for pain during walking improved significantly at week 5 and month 6 (*p =* 0.0087 and *p =* 0.0049, respectively), whereas VAS score at rest showed a difference compared to placebo, but not significant [31]. In an RCT, IA-HA injection showed a significant reduction in Lequesne index at week 5 vs. placebo (from 13.57 ± 1.88 to 7.94 ± 2.53, *p <* 0.01), in addition to improving total workload of knee flexion and extension (*p <* 0.01). IA-HA posology consisted of a weekly injection for a total of five injections [32].

Significant favorable differences from baseline were noted in the HA-treated group for the VAS scale and Lequesne index at week 4. However, the HA-treated group received an IA injection a week for 3 weeks (4 injections) [33]. Although using a different index to measure pain, stiffness, and disability than the Lequesne index, Petrella et al. (2006) showed, in a double-blinded RCT, a significant improvement in WOMAC scores for knee pain in the HA-treated group at week 3 (*p* < 0.05), with no further difference at weeks 6 and 12. Pain assessment using the VAS scale showed similar improvement between the placebo and HA-treated group, with no further differences at weeks 6 and 12. An interesting observation resides in the improvement in the knee joint’s range of motion in our study, whilst there was no improvement noted in the range of knee joint motion at weeks 6 and 12 compared to baseline (*p* = 0.89 and 0.59, respectively) in Petrella et al. (2006) trial [34].

A similar study found an improvement in WOMAC and VAS score for resting pain at week 4 (*p <* 0.05) in the HA and placebo-treated group, NSAIDs, misoprostol, and HA-treated group, and NSAIDs and placebo-treated group compared to baseline. At week 12, the aforementioned groups had no significant improvement [35]. In a prospective study, Weinhart (2008) showed a reduction in pain severity score at different stages (at night, at rest, starting to walk, and exertion pain) after five injections and 4 weeks after therapy, simultaneously decreasing Lequesne index summation score when measured at the same periods (3.45 points decrease after five injections and 5.81 points decrease at 4 weeks post-therapy from baseline measurements) [36].

A systematic review of overlapping meta-analyses that included 20,049 patients from 14 meta-analyses (13,698 receiving IA HA, 255 receiving NSAIDs, 294 receiving IA corticosteroids and 5702 receiving IA placebo) found that IA HA improved pain and function, while no clinically relevant differences regarding efficacy when compared with NSAIDs were found, whereas clinical benefit of IA HA were greater at 5 to 13 weeks and persisted up to 26 weeks, concluding that IA HA injections represent a viable alternative in patients with early knee OA [37]. In a Cochrane review, Bellamy et al. (2006) evaluated 76 trials, in which follow-up periods varied between the day of the last injection and 18 months. A total of 40 trials investigated whether the difference between HA and placebo exists. The pooled analyses of the effects of HA vs. placebo support the efficacy of IA HA administration, especially at weeks 5 and 13 post-injection, showing a percentage improvement from baseline of 28–54% for pain parameter and 9–32% for function. When compared to NSAIDs, efficacy was similar, whereas HA compared to IA corticosteroids favors IA HA, especially regarding long-term effects [38]. The results of this confirmatory study are consistent with the findings of previous studies and support the benefit/risk ratio of IA HA and HA-CS injections in OA patients.

Viscosity (η) is a parameter that can only be measured for bodies in a fluid (liquid) state, expressing the ability of the fluid to resist the sliding of two adjacent layers of its mass, during the movement of the mass of fluid through flow. In the case of products with intra-articular application, structurally fluid systems at ambient temperature, the fluid state occurs when the system is sheared with a force that exceeds the stress value called yield threshold (τ_0_), a parameter dependent on the system and its characteristics. After this threshold, the flow behavior is determined by the resistance that the fluid exhibits to the shear force (speed gradient, shear rate) applied to it, a property that is expressed by the value of the tangential stress (τ). Tangential flow stress (τ) and viscosity (η) are interdependent parameters that vary directly or inversely proportionally, depending on the shear flow behavior, and each of these two (measurable) parameters can be used to evaluate the consistency of a product that is administered intra-articularly and comparison with the properties of synovial fluid from a healthy person. The rheological behavior of structurally viscous systems is characterized by two values (parameters): the yield point - τ_0_ (N/m^2^) and, respectively, the plastic viscosity -η (mPa·s). The properties of the synovial fluid are different when it comes to a healthy person, and when it comes to a condition such as osteoarthritis, not only the amount of hyaluronic acid is changed, but also the visco-elastic properties [29].

The rheological study demonstrates the distinctive viscoelastic properties of the SH (20 mg/mL) and CS (30 mg/mL) formulation compared to the control (20 mg/mL SH only). This finding suggests that the addition of chondroitin sulfate (CS) to sodium hyaluronate (SH) significantly enhances and improves the viscoelastic properties, particularly at higher shear frequencies (e.g., running vs. walking). The application of oscillatory frequency provided valuable insights into the structure–property relationship of the biomatrix solution. The observed shift in the crossover frequency towards lower frequencies upon CS addition indicates larger relaxation times, suggesting a more entangled network structure. This aligns with the observed plateau in the elastic modulus (G′) at the entanglement region, signifying a transition towards more elastic solid-like behavior. Furthermore, the robust shear thinning response of the SH-CS formulation at high shear rates implies shear-dependent behavior, potentially beneficial for in vivo applications. The relaxation times in the range of 5–10 s suggest a balance between viscous and elastic components, while the flattening of G′ beyond the crossover frequency and dominance of G″ within a wider range indicate a viscoelastic response dominated by the elastic component. Rheological analysis showed that chondroitin sulfate markedly increases the viscosity of HA solutions under physiological conditions, giving further insights into the physiological role of chondroitin sulfate and chondroitin sulfate proteoglycans in extracellular matrices and body fluids.

Limitations regarding the present study include a small number of participants (21), and a lack of a prolonged follow-up period to assess the clinical outcome parameters in the long term. To overcome these limitations, further studies ought to be conducted on a larger population and a longer follow-up period to assess performance in the long term.

There are many products on the market in Europe that are administered intra-articularly, but the vast majority only focus on the different compositions and different molecular weights of hyaluronic acid. The complex biomatrix that includes chondroitin sulfate and the achievement of viscoelastic properties similar to the synovial fluid of a healthy patient proves to be a personalized medication that could increase the patient’s degree of satisfaction after administration [39].

In future studies, we propose to evaluate through a wide post-market questionnaire the degree of patient satisfaction with a return to daily activities/work correlated with the assessment of the attending physician, after one cycle of treatment with an HA-CS combination. Both the patient’s perception and the specialist’s medical judgment are important for evaluating the patient’s degree of satisfaction. The extremely promising data of this product require further investigation with respect to clinical implications under current medical practice in knee osteoarthritis patients.

## 5. Conclusions

IA-HA is an established therapy for OA treatment. The first product of sodium hyaluronate was approved by the FDA in 1997. Even though hyaluronic acid administered intra-articularly has been used since 1987 in Europe and Japan, through the proposed study we wanted to come up with eloquent data about the effectiveness of hyaluronic acid and chondroitin sulfate administered for knee osteoarthritis. The present study was designed to support data regarding the positive clinical outcomes of HA and CS viscoelastic solutions for IA injections in patients with OA. Based on our study results, the one-time injection proved to significantly alleviate OA symptoms until 24 weeks after injection, which confirms its suitability as a viscoelastic supplement or a replacement for synovial fluid in human knee joint osteoarthritis. No adverse events have been noted during the study period.

By adding chondroitin sodium sulfate at a ratio of 3:2 to natural hyaluronic acid supported natural and physical crosslinking of sodium hyaluronate providing mechanical robustness, improved rheological properties, simultaneously with preserving biocompatibility and biodegradability of the sodium hyaluronate native polymer. At the same time, better resistance to enzymatic degradation and free radicals could be expected from the synergic combination of sodium hyaluronate and chondroitin sodium sulfate.

## Figures and Tables

**Table 1:** Patient’s demographics.

Gender	4 male (19.1%)
	17 female (80.9%)
Age	61.8 ± 8.2 years
Mean age of OA	7.9 ± 3.67 years

**Table 2:** Pearson’s coefficient of skewness (average ± SD).

Timeframe (Weeks)	Pearson’s Coefficient of Skewness
Baseline	169.82 ± 35.41
6	200.56 ± 39.98
12	223.55 ± 42.71
24	231.21 ± 44.89

**Table 3:** Rheological parameters for HH commercial batch.

Temperature	25 ± 0.01 °C		37 ± 0.01 °C	
Reference frequency	0.5 ^a^ Hz	2.5 ^b^ Hz	0.5 ^a^ Hz	2.5 ^b^ Hz
Elastic modulus (G′) (Pa)	271.6	478.3	174.4	198.8
Viscous modulus (G″) (Pa)	208.7	394.8	149.8	181.7
Cross-over frequency (Hz)	0.096 ^c^		0.171 ^c^	
Relaxation time (s)	10.4		5.8	
Elasticity (%)	60.9	70.6	58.2	68.5

^a^—representative for walking, and ^b^—2.5 Hz representative for running; ^c^—the frequency at which G′ = G″.

**Table 4:** Comparative rheological parameters for HH vs. Control at 25 ± 0.01 °C.

Viscosupplement	HH		Control	
Reference frequency	0.5 ^a^ Hz	2.5 ^b^ Hz	0.5 ^a^ Hz	2.5 ^b^ Hz
Elastic modulus (G′) (Pa)	271.6	478.3	82.6	208.0
Viscous modulus (G″) (Pa)	208.7	394.8	91.4	136.4
Cross-over frequency (Hz)	0.096 ^c^		0.660 ^c^	
Relaxation time (s)	10.4		1.5	
Elasticity (%)	60.9	70.6	47.5	60.4

^a^—0.5 Hz representative for walking, and ^b^—2.5 Hz representative for running; ^c^—frequency at which G′ = G″.

## Data Availability

Data are contained within the article.
